# Removal of fungal ball from the jaws by lefort i osteotomy: Difficulty in diagnosing patients with chronic sinusitis

**DOI:** 10.4317/jced.57692

**Published:** 2021-03-01

**Authors:** Andressa-Bolognesi Bachesk, Verônica-Ramos de Souza, Carla-Militão Ricken, Ricardo-Augusto-Gonçalves Pierri, Angelo-José Pavan

**Affiliations:** 1 DDS, Resident in Oral and Maxillofacial Surgery at State University of Maringá – Maringá – PR – Brazil; 2 DDS, Intern in Oral and Maxillofacial Surgery at State University of Maringa – Maringá – PR – Brazil; 3 Undergraduate student in Dentistry at State University of Maringa – Maringa – PR - Brazil; 4 PhD, Professor of Oral and Maxillofacial Surgery at State University of Maringá – Maringá – PR – Brazil

## Abstract

Aspergillosis, aka fungal ball (FB), is classified as a type of non-invasive fungal rhinosinusitis, which usually occurs unilaterally in the maxillary sinus of an immunocompromised patient. Its diagnosis is complex and depends on the association between clinical, imaging, and histopathological exams. There are many treatments for fungal infections of the paranasal sinuses, so early diagnosis is extremely important to determine the appropriate treatment. This paper reports an unusual clinical case of aspergillosis present bilaterally inside the maxillary sinuses of a healthy patient, associated with mucous retention cysts, whose imaging exams and transnasal endoscopy were not sufficient to precisely identify the lesion. Its diagnosis and definitive treatment were obtained only after orthognathic surgery and integration between a multidisciplinary team.

** Key words:**Aspergillosis, communicable diseases, fungal ball, infectious disease, orthognathic surgery.

## Introduction

The Fungal Ball (FB), or aspergillosis of the maxillary sinuses, is classified as a non-invasive fungal infection caused mainly by the species Aspergillus fumigatus (1,2). This type of sinusitis presents itself as an extra-mucous dense tangle of hyphae, which are in different stages of decomposition. Its etiopathogenesis is uncertain, with functional obstruction of the maxillary ostium and odontogenic sources (such as endodontic treatment and bucosinusal communication), possible causes reported in the literature (1).

The infection occurs in the maxillary sinuses of immunocompromised patients and is usually exclusive (without associated pathologies) and unilateral, with rare bilateral lesions (1,3,4). Clinically, it can be asymptomatic or simulate chronic rhinosinusitis events, with nasal secretion and/or congestion, chronic sinus pain, headache, and hyposmia (5,6). Radiographically, it displays a radiopaque image, which resembles a foreign body, associated with the veiling of the involved maxillary sinus (4,7). Differential diagnoses include anthrolytes, mucoceles, allergic sinusitis, and cholesterol granuloma, and the definitive diagnosis is obtained only after histopathological examination (8). Therefore, due to the difficulty of obtaining an accurate diagnosis, this condition is often unsatisfactorily addressed (2).

The treatment of this pathology is surgical, either by nasal endoscopic surgery or by direct access to the lesion. The complete removal of the fungal ball is sufficient to allow natural sinus drainage; however, the use of antifungal medication is also indicated (4,9,10). The objective of this study is to report a clinical case of fungal ball associated with mucous retention cysts, present bilaterally in the maxillary sinuses of a healthy patient, which ended up developing epiphora. This is an unusual case, which, due to the difficulty in early identification, was successful only due to diagnosis and treatment during the intraoperative period of orthognathic surgery.

## Case Report

**Figure 1:**
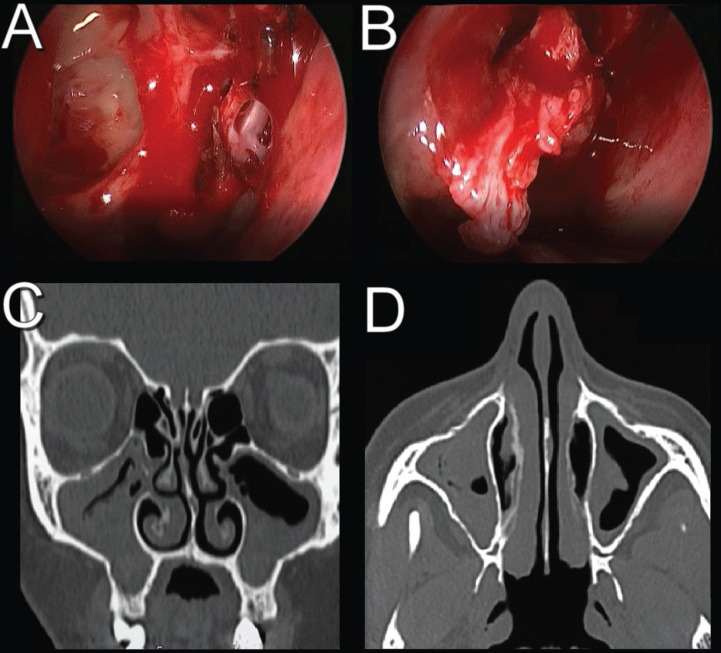
Initial complementary exams. A and B) Sequence of trans-surgical images of transnasal endoscopy; C) Coronal section of computed tomography; D) Axial section of computed tomography.

**Figure 2:**
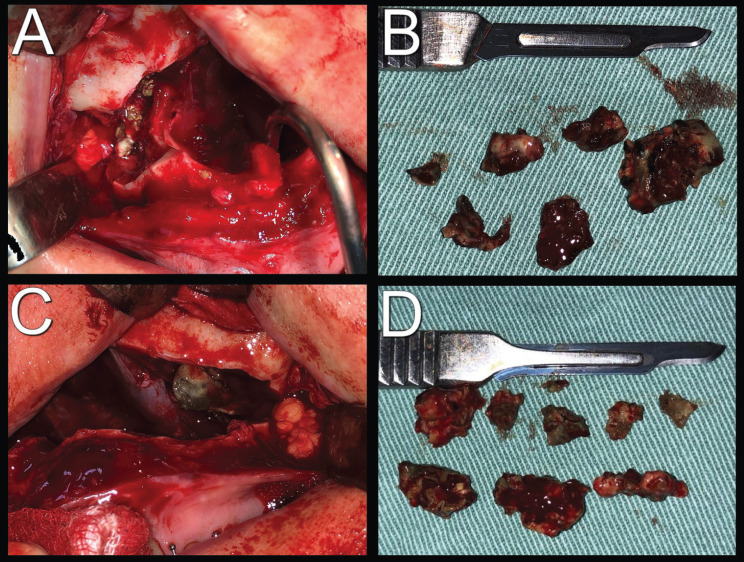
Trans-surgical images showing fungal balls after Lefort I osteotomy. A) Infected right maxillary sinus; B) Lesions removed from the right maxillary sinus; C) Infected left maxillary sinus; D) Lesions removed from the left maxillary sinus.

**Figure 3:**
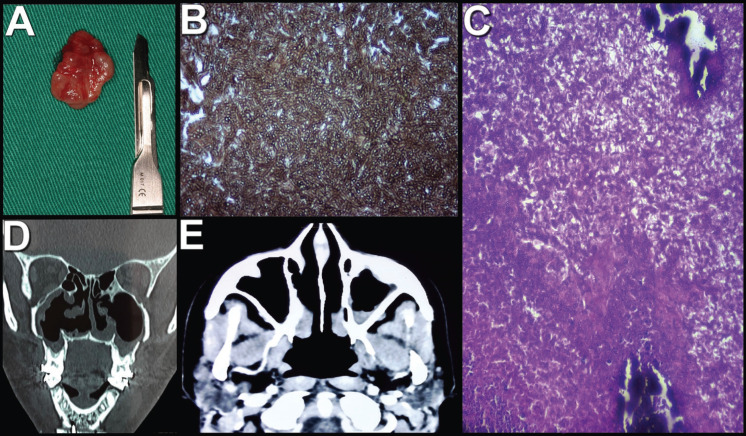
Diagnosis and follow-up. A) Trans-surgical image after enucleation of the mucous retention cyst; B) Histological section of the material collected by special Grocott-Gomori’s Methenamine Silver stain, positively evidencing the presence of thick hyphae - the main etiological agent was Aspergillus sp; C) Histological section with Hematoxylin-Eosin (HE) staining showing, in the midst of fibrin and cell debris, moderate amount of neutrophils and calcification areas. Presence of thick fungal hyphae; D) Coronal section of computed tomography; 3E) Axial section of computed tomography with soft tissue window.

A 42-year-old female patient was referred to the Oral and Maxillofacial Surgery team, with an indication for orthognathic surgery. During anamnesis, she reported a history of chronic sinusitis, which triggered headaches, facial pain, constant episodes of rhinorrhea, and breathing difficulties. The patient also reported having undergone treatment with an otorhinolaryngologist for six years. During this period, several antibiotic prescriptions and nasal endoscopy exams were performed (Fig. 1A,B), however, with no results. On tomographic examination, bilateral opacification of the maxillary sinuses was found (Fig. 1C,D). Therefore, an endodontic assessment of the maxillary teeth was requested, which found no signs of pulp involvement. Orthognathic surgery planning was employed, as it was a chronic clinical condition, and the patient had an indication for this surgical procedure. Thus, during the intraoperative period, after Lefort I downfracture, the presence of dark and foamy masses was found, which occupied a quarter of the maxillary sinuses, bilaterally (Fig. 2). In addition, a mucus-like soft tissue was observed on both floors of these paranasal sinuses (Fig. 3A). Based on the findings, the hypotheses of fungal balls and mucous retention cysts were considered. Progress was made with total curettage of the maxillary sinuses.

The collected material was sent for histopathological analysis, which confirmed the diagnosis of fungal balls (predominantly Aspergillus sp) (Fig. 3B,C) and mucous retention cysts. After the surgical procedure, the patient obtained a significant improvement in her breathing capacity, in addition to the reduction of sinus symptoms, and was subsequently referred to an infectologist and an otorhinolaryngologist for assessment and additional treatment. After a 1-month follow-up post-surgery, the patient developed an epiphora. With this, she was referred to the ophthalmologist, who opted for Dacryocystorhinostomy, with ductal clearance by a possible fungal ball. The patient used Itraconazole 200mg daily for 07 months and is in the postoperative period of 02 years, with significant regression of clinical and imaging signs and symptoms (Fig. 3D,E).

## Discussion

The sinus fungal ball, usually found in the maxillary sinus, can develop via respiratory or odontogenic routes (1). From a clinical point of view, aspergilloma is generally underestimated, because the infection only becomes symptomatic after a long period of fungal contamination (11). Some of the reported symptoms are nasal obstructions, purulent nasal discharge, hyposmia, and facial pain. Less common signs include seizures, epistaxis, proptosis, fever, cough, and blurred vision (12). In our case, the patient had a headache, facial pain, rhinorrhea, and breathing difficulties, which lasted for six years.

Aspergillosis is generally unilateral, with a prevalence of approximately 94% (13). In addition, when it occurs, the disease is mostly exclusive, with rare episodes associated with other pathologies (8). These facts make our case unusual since fungal clusters were found in both bilateral maxillary sinuses, associated with mucous retention cysts. Despite the particularities of this case, studies show that more than 10% of patients who have chronic sinusitis also have aspergilloma, especially in the maxillary sinus (10,11). Therefore, although the fungal ball is the most common form of non-invasive fungal rhinosinusitis observed in clinical practice (14), its diagnosis is often difficult and inadequate (8).

Computed tomography (CT) is an important complementary exam to assist in the differential diagnosis, however, alone, it is not enough to generate the definitive result. Hypotheses such as anthrolytes, osteomas, mucoceles, B cell lymphoma, squamous cell carcinoma, cystic adenoid carcinoma, and inflammatory myofibroblastic tumors are lesions of possible similarities in imaging exams (15). Authors report that nasal endoscopy should be performed in all patients who have nasosinusal complaints, however, this may not be sufficient for the diagnosis of the fungal ball. This exam is not specific and presents variable findings such as normal mucosa, discharge of purulent secretion, edema, and polyps (16). This fact supports the presented case since the patient had undergone nasal endoscopy, which was insufficient to identify the lesion, delaying the correct diagnosis in years.

As for the treatment of the fungal ball, the surgical techniques of Caldwell-Luc and endoscopic techniques may be used, aiming at the complete removal of the infection (4,10). In our case, as the patient was referred with satisfactory orthodontic preparation for orthognathic surgery, it was decided to perform this corrective surgery simultaneously with the diagnosis of sinusitis. This is because the downfracture of the maxilla, after Le Fort I osteotomy, allows wide access and direct view of the maxillary sinuses. Therefore, through this technique, it was possible to perform a full curettage of both sinuses, promoting the treatment of facial anomaly and infectious condition, in a single surgical procedure. However, in most situations, where this indication is not feasible, the performance of exploratory surgery, as by the technique of Caldwell Luc is shown to be a viable alternative, technically simple and inexpensive (4,10). This is because it allows the diagnosis, by direct visualization of the lesion and obtaining material for biopsy, and also the treatment, because through the bone window it is possible to remove the pathology from inside the maxillary sinuses.

Although there are reports in the literature of orbital impairment by aspergillosis, the development of epiphora due to nasolacrimal duct obstruction is a rare symptom, and when it occurs, it tends to be due to progression of aspergillosis to the sphenoid sinuses and in cases of invasive arpergillosis (17). In the reported case the patient presented noninvasive aspergillosis with tomographic images of the clean sphenoid sinuses. The ductal obstruction by to a possible fungal ball was considered due to the trans-surgical clinical characteristics and previous history, and therefore, the importance of the multidisciplinary approach of requesting the assistance of the ophthalmologist is noted.

It is worth mentioning the importance of early diagnosis and the correct classification of fungal sinusitis since the management and prognosis are very different (13). Therefore, providing an effective therapy is crucial to achieve a better result. With this in mind, we observe the importance of the exchange of knowledge between a multi-professional team. Because only after integration between surgery, otorhinolaryngology, and infectology, the correct diagnosis was obtained and proper treatment was employed with the assistance of the ophthalmologist. In conclusion, the early diagnosis of the fungal ball, although complex, is extremely important to guarantee the appropriate treatment for the patient, reducing his/her exposure to unnecessary procedures and incorrect medication. The interaction of a multidisciplinary team is essential to achieve a good result and to avoid the possibility of underestimation and worsening of the injury. Effective treatment for this type of infection depends on surgical debridement and, in some cases, associated antifungal therapy.
